# Supplementary material for: Stress and Nasal Allergy: Corticotropin-Releasing Hormone Stimulates Mast Cell Degranulation and Proliferation in Human Nasal Mucosa
Source: Int J Mol Sci. 2021 Mar 9;22(5):2773. doi: 10.3390/ijms22052773 (PMC7967145; doi:10.3390/ijms22052773)
Supplement: Supplementary file 1 [file ijms-22-02773-s001.pdf]

Supplementary Materials

Stress and Nasal Allergy: Corticotropin-Releasing Hormone Stimulates Mast Cell Degranulation and Proliferation in Human Nasal Mucosa

Mika Yamanaka-Takaichi [1,†], Yukari Mizukami [1,†], Koji Sugawara [1,†,*], Kishiko Sunami [2], Yuichi Teranishi [2], Yukimi Kira [3], Ralf Paus [4,5,6] and Daisuke Tsuruta [1]

[1] Department of Dermatology, Osaka City University Graduate School of Medicine, Osaka, Japan; mikay0115@gmail.com (M.Y.-T.); cebu-yuka@hotmail.co.jp (Y.M.); kojilaminin@yahoo.co.jp (K.S.); dts211@gmail.com (D.T.)
[2] Department of Otolaryngology and Head and Neck Surgery, Osaka City University Graduate School of Medicine, Osaka, Japan; kishiko@med.osaka-cu.ac.jp (K.S.); yteranishi@med.osaka-cu.ac.jp (Y.T.)
[3] Department of Research Support Platform, Osaka City University Graduate School of Medicine, Osaka, Japan; yukimi@med.osaka-cu.ac.jp
[4] Dr. Phillip Frost Department of Dermatology & Cutaneous Surgery, University of Miami Miller School of Medicine, Miami, FL, USA; rxp803@med.miami.edu
[5] Centre for Dermatology Research, University of Manchester, and NIHR Manchester Biomedical Research Centre, Manchester, UK; ralf.paus@manchester.ac.uk
[6] Monasterium Laboratory, Münster, Germany
* Correspondence: kojilaminin@yahoo.co.jp; Tel.: +81-6-6645-3826
† These authors contributed equally to this work

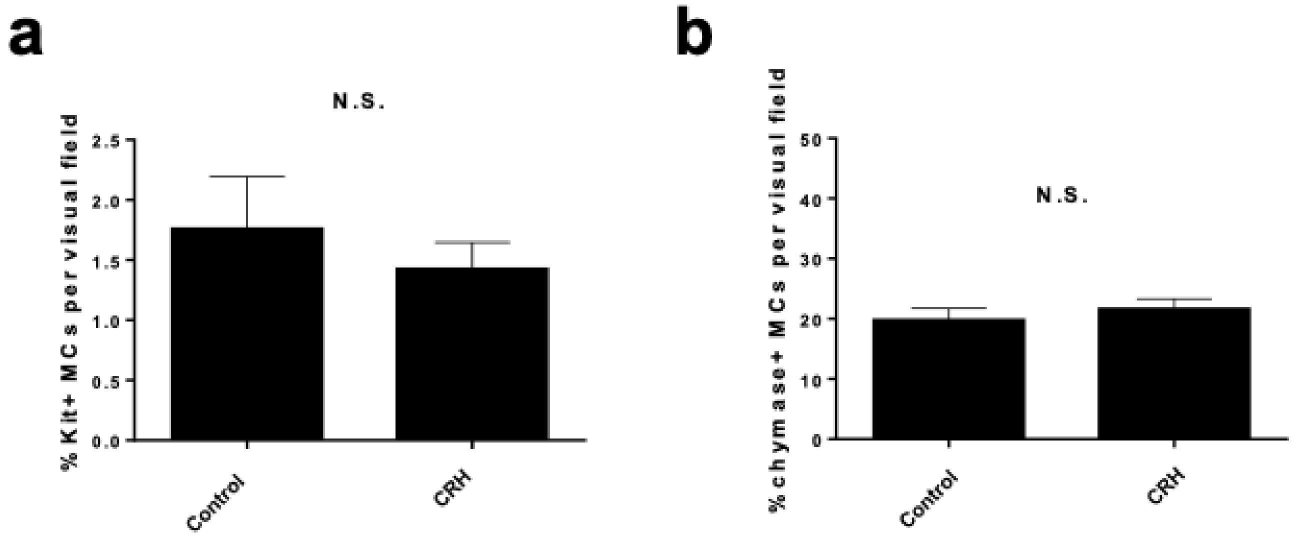

Figure S1. Quantitative immunohistomorphometry of c-Kit+ and chymase+ cells in the lamina propria of NPs. CRH did not alter the number of c-Kit+ or chymase+ hM-MCs in NPs. (a) Quantitative immunohistomorphometry of c-Kit+ cells. n = 5. (b) Quantitative immunohistomorphometry of chymase+ cells. n = 7. Error bars indicate the standard error of the mean (SEM). N.S. = not significant. MC, mast cell; hM-MCs, human nasal mucosa MCs; CRH, corticotropin-releasing hormone; NPs, nasal polyps.

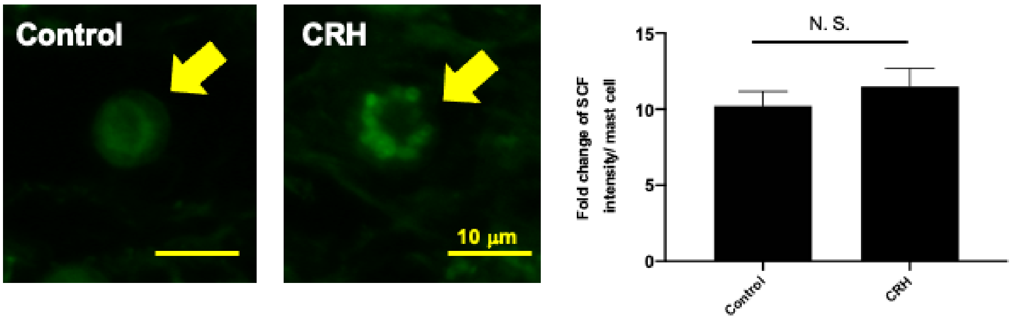

Figure S2. SCF expression within the tryptase/SCF double+ hM-MCs was higher in the CRH-treated NP group, but no significant differences were shown. An arrow denotes SCF+ hM-MCs. n = 6; scale bar = 10 μm. Error bars indicate SEM. N.S. = not significant. MC, mast cell; SCF, stem cell factor; hM-MCs, human nasal mucosa MCs; CRH, corticotropin-releasing hormone; NP, nasal polyp.

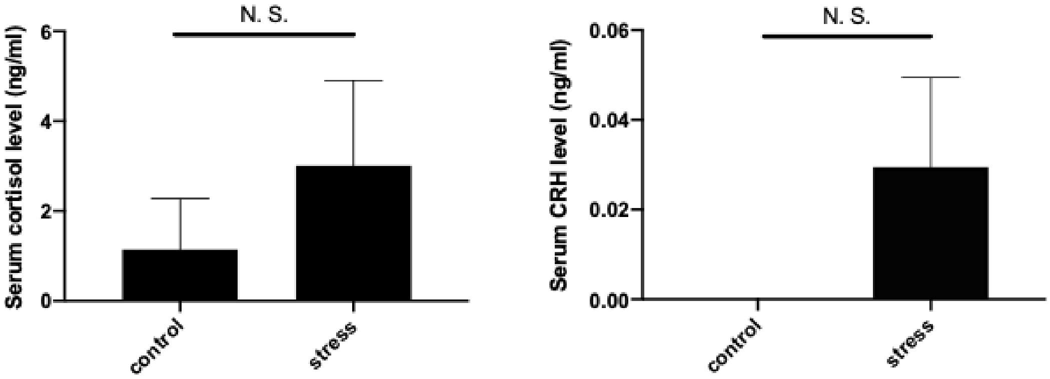

Figure S3. Effect of restraint stress on serum cortisol and CRH levels. Control group, n = 4 (without stress); stress group, n = 4. Error bars indicate SEM. N.S. = not significant. CRH, corticotropin-releasing hormone.

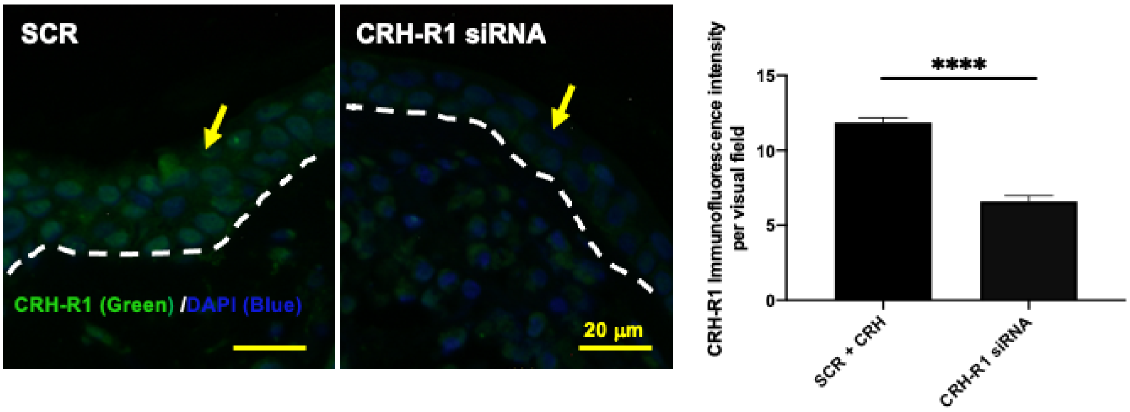

Figure S4. CRH-R1 immunoreactivity in NP epithelium with CRH-R1 siRNA-treated and control siRNA-treated human NPs. Yellow arrows indicate positive CRH-R1 immunoreactivity. n = 4; scale bar = 20 μm. Error bars indicate SEM. ****P < 0.0001. CRH, corticotropin-releasing hormone; CRH-R1, CRH receptor type 1; NP, nasal polyp; SCR, scrambled small interfering RNA (siRNA)–treated NPs; CRH-R1 siRNA, CRH-R1 siRNA– treated NPs; DAPI, diamidino-2-phenylindole.

Table S1. Patient list for the NPs used in the study.

Patient	Age (years)	Sex	Asthma	Aspirin in-Tolerance	Allergy	Serum IgE	Co-Medication at Time of Surgery	Co-Morbidities	Experiments
1	58	F	○	–	contrast medium	–	oral antihistamine, inhaled corticosteroid	–	TB, tryptase/CRH-R1 IF, tryptase/CRH-R2 IF, c-Kit/CRH-R1 IF, chymase IHC, tryptase/Ki67 IF, SCF/AE1/3 IF, tryptase/SCF IF
2	33	M	○	–	mite	–	oral antihistamine	–	TB, tryptase/CRH-R1 IF, tryptase/CRHR-2 IF, c-Kit/CRH-R1 IF, chymase IHC, tryptase/Ki67 IF, tryptase/PCNA IF, tryptase/TUNEL IF, tryptase/SCF IF
3	68	M	○	○	NSAIDs	high	antibiotics, inhaled corticosteroid	–	CRH IHC, TB, tryptase IHC, c-Kit IHC, tryptase/Ki67 IF, SCF/AE1/3 IF, tryptase/SCF IF
4	80	F	○	–	pollen	high	oral antihistamine, inhaled corticosteroid	HLD, HT	CRH IHC, TB, tryptase/CRH-R1 IF, tryptase/CRHR-2 IF, c-Kit/CRH-R1 IF, tryptase IHC, c-Kit IHC, chymase IHC, tryptase/PCNA IF, tryptase/TUNEL IF, SCF/AE1/3 IF, tryptase/SCF IF
5	61	M	–	–	pollen	normal	oral antihistamine, antibiotics	alcoholic hepatitis, HT	CRH IHC, tryptase/CRH-R1 IF, tryptase/CRHR-2 IF, tryptase IHC, c-Kit IHC, chymase IHC, tryptase/PCNA IF, tryptase/TUNEL IF, tryptase/SCF IF
6	64	M	○	–	–	high	oral antihistamine, inhaled corticosteroid	HLD, HT, prostatic hypertrophy	CRH IHC
7	69	F	–	–	–	normal	oral antihistamine, antibiotics	HT, depression	CRH-R1 gene knockdown
8	50	F	–	–	–	normal	–	anemia	TB, tryptase/CRH-R1 IF, tryptase/CRH-R2 IF, c-Kit/CRH-R1 IF, chymase IHC, tryptase IHC, tryptase/Ki67 IF, tryptase/TUNEL IF, tryptase/PCNA IF, SCF/AE1/3 IF, tryptase/SCF IF
9	26	F	–	–	–	–	antibiotics	premature atrial-contraction	tryptase/Ki67 IF, tryptase/CRH-R1 IF
10	50	M	○	–	cat	high	antibiotics	hyperthyroidism	tryptase/Ki67 IF, tryptase/CRH-R1 IF
11	48	F	○	–	–	–	antibiotics, inhaled corticosteroid	–	CRH-R1 gene knockdown
12	49	F	–	–	–	–	–	HT, anemia	CRH-R1 gene knockdown
13	48	M	○	○	aspirin	normal	inhaled corticosteroid	–	CRH-R1 gene knockdown
14	39	M	–	–	–	–	oral antihistamine	HT, hepatic dysfunction	tryptase IHC, c-Kit IHC, chymase IHC
15	73	M	○	○	NSAIDs	–	–	HT, reflux esophagitis	tryptase IHC, c-Kit IHC, chymase IHC

Total serum IgE level; normal (150-300 UI/ml) and high (> 300 UI/ml). F, female; M, male; NSAIDs, non-steroidal anti-inflammatory drugs; HLD, hyperlipidemia; HT, hypertension; TB, toluidine blue histochemistry; IF, immunofluorescence; IHC, immunohistochemistry; CRH, corticotropin-releasing hormone; CRH-R1, CRH receptor type 1; CRH-R2, CRH receptor type 2; PCNA, proliferating cell nuclear antigen; TUNEL, terminal deoxynucleotidyl transferase-mediated dUTP nick end-labeling; SCF, stem cell factor.

Table S2. List of all immunostainings and relevant details.

Antigens	Type	Section	Antigen Retrieval/Fixation	1st Detection System	2nd Detection System	Counter Staining
CRH	IHC	paraffin	Tris-EDTA	ABC-AP, SIGMAFAST		hematoxylin
CRH-R1	IF	paraffin	sodium citrate	IF, Alexa 488		DAPI
CRH-R2	IF	paraffin	sodium citrate	IF, Alexa 488		DAPI
CRH-R1/Tryptase	IF	paraffin	sodium citrate	IF, Alexa 488	IF, Alexa 594	DAPI
CRH-R1/c-Kit	IF	paraffin	Tris-EDTA	IF, Alexa 488	IF, Alexa 594	DAPI
CRH-R2/Tryptase	IF	paraffin	sodium citrate	IF, Alexa 488	IF, Alexa 594	DAPI
Tryptase	IHC	paraffin	Tris-EDTA	ABC-AP, SIGMAFAST		hematoxylin
C-Kit	IHC	paraffin	Tris-EDTA	ABC-AP, SIGMAFAST		hematoxylin
Chymase	IHC	paraffin	Tris-EDTA	ABC-AP, SIGMAFAST		hematoxylin
Tryptase/PCNA	IF	paraffin	sodium citrate	IF, Alexa 488	IF, Alexa 594	DAPI
Tryptase/Ki67	IF	paraffin	sodium citrate	IF, Alexa 488	IF, Alexa 594	DAPI
SCF	IF	paraffin	Proteinase K	IF, Alexa 488		DAPI
SCF/AE1/3	IF	paraffin	Proteinase K	IF, Alexa 488	IF, Alexa 594	DAPI
SCF/Tryptase	IF	paraffin	sodium citrate	IF, Alexa 488	IF, Alexa 594	DAPI

ABC-AP, avidin-biotin complex-alkaline phosphatase; DAPI, diamidino-2-phenylindole; Alexa 488, Alexa flour 488; Alexa 594, Alexa flour 594; EDTA, ethylenediaminetetraacetic acid; IF, immunofluorescence; SIGMAFAST, Fast Red TR/Naphthol AS-MX tablets; CRH, corticotropin-releasing hormone; CRH-R1, CRH receptor type 1; CRH-R2, CRH receptor type 2; PCNA, proliferating cell nuclear antigen; SCF, stem cell factor.
